# Neural Coding for Effective Rehabilitation

**DOI:** 10.1155/2014/286505

**Published:** 2014-09-02

**Authors:** Xiaoling Hu, Yiwen Wang, Ting Zhao, Aysegul Gunduz

**Affiliations:** ^1^Interdisciplinary Division of Biomedical Engineering, The Hong Kong Polytechnic University, Hung Hom, Kowloon, Hong Kong; ^2^Qiushi Academy for Advanced Studies, Zhejiang University, Zhejiang 310027, China; ^3^Howard Hughes Medical Institute, Janelia Farm Research Campus, Ashburn, VA 20147, USA; ^4^J. Crayton Pruitt Family Department of Biomedical Engineering, University of Florida, Gainesville, FL 32611, USA

## Abstract

Successful neurological rehabilitation depends on accurate diagnosis, effective treatment, and quantitative evaluation. Neural coding, a technology for interpretation of functional and structural information of the nervous system, has contributed to the advancements in neuroimaging, brain-machine interface (BMI), and design of training devices for rehabilitation purposes. In this review, we summarized the latest breakthroughs in neuroimaging from microscale to macroscale levels with potential diagnostic applications for rehabilitation. We also reviewed the achievements in electrocorticography (ECoG) coding with both animal models and human beings for BMI design, electromyography (EMG) interpretation for interaction with external robotic systems, and robot-assisted quantitative evaluation on the progress of rehabilitation programs. Future rehabilitation would be more home-based, automatic, and self-served by patients. Further investigations and breakthroughs are mainly needed in aspects of improving the computational efficiency in neuroimaging and multichannel ECoG by selection of localized neuroinformatics, validation of the effectiveness in BMI guided rehabilitation programs, and simplification of the system operation in training devices.

## 1. Introduction

Neurological rehabilitation usually is a long-term process for patients suffering from trauma or disorders of the nervous system. With the growth of the ageing population across the world, the number of patients with degenerative (e.g., Parkinson disease, amyotrophic lateral sclerosis (ALS)) and vascular disorders (e.g., stroke) has increased substantially. In fact, stroke, a cerebrovascular accident, has been identified as the leading cause of adult disability [1, 2]. Providing long-term and effective rehabilitation service has been a grand challenge in many countries and has created pressure to current medical care systems [3]. Neural coding, a technology for interpretation of functional and structural information of the nervous system, has contributed a lot to the advancements in neurological rehabilitation.

Successful neurological rehabilitation firstly depends on the accurate diagnosis of the underlying pathology, its anatomical foci, and the effects on functional networks and structural connections. The advancements in volumetric neuroimaging technology now allow us to visualize neural networks in detail from macroscale to microscale levels. For instance, functional magnetic resonance imaging (fMRI) is now commonly used clinically for diagnosis on the cerebral network reorganization after stroke, that is, the macroscale imaging, and with the application of high-resolution optical microscopy, the structure and the dynamic connection among a group of neurons could be revealed, that is, microscale imaging [4]. Neural imaging provides not only the diagnostic information, but also the mechanism or theoretical support for designing optimal rehabilitation therapy as an evaluation tool.

Effective treatment is the second important component in the rehabilitation. The traditional physical and occupational therapies are mainly conducted by human therapists, who can interact with a patient and support him/her to complete the desired training tasks. However, with the shortage of the rehabilitation professionals and the growing population of the patients, assistive rehabilitation devices (e.g., rehabilitation robots) are in great demand. The design of rehabilitation devices that can interact with the patients is based on the identification of voluntary motor intention of a user. Brain-machine interface (BMI), sometimes termed as brain-computer interface (BCI), is a technique that has been explored to decode such voluntary motor intention from brain signals, for example, electroencephalography (EEG) and electrocorticography (ECoG), which are the neural potentials detected from the scalp and the brain, respectively [5, 6]. ECoG, also known as intracranial EEG (iEEG), refers to neural recordings from the cortical surface through a surgical incision to the skull. The detection of ECoG is invasive with the electrode array directly attached to the brain; however, ECoG signals have much higher spatial and temporal resolution than the scalp EEG. BMIs with ECoG detection have been mainly investigated in animal models, for example, monkeys, for possible interaction with external systems, such as a computer game, or even a prosthetic robot [7, 8]. The animal BMI studies paved the road to the application of ECoG BMI to human beings for rehabilitation purposes. BMI technique is important in designing rehabilitation devices for severely paralyzed patients, whose limb motions are hardly to be detected. Besides the neural signals detected from the brain, muscular electricity, that is, electromyography (EMG), also has been used to explore the neural instructions to the muscles. In comparison with neural or neuronal signals, EMG has focalized resolution on individual muscles and relatively higher amplitudes detected noninvasively. Therefore, EMG is also a favorable biosignal in controlling rehabilitation devices for patients with residual muscle functions [9, 10].

The third important component in neural rehabilitation is the quantitative evaluation during and after physical training. Rehabilitative treatment is a long-term intervention that usually lasts for years, during which the development of diseases or the progress of recovery needs to be monitored for the adaptation of treatment programs. However, most of the assessment tools used clinically are subjective based on the observation of practitioners, such as the Fugl-Meyer Assessment [11] for evaluation of upper limb motor function and the Modified Ashworth Scores for assessing muscular spasticity [12]. Due to the lack of manpower in rehabilitation industry, even the subjective evaluations are sparse in most of clinical services currently. New methods are needed for quantitative and long-term assessment on the rehabilitation progress and the posttraining follow-ups. Taking advantage of the neural coding technique, it is possible that training devices also can act as evaluation systems. In this review, we summarized the latest breakthroughs in neural coding with the potential application for more effective rehabilitation on neural network imaging and neural informatics in the cortical areas of monkey during dynamic limb motions; we also reviewed the achievements in neural coding by electrocorticographic interpretation in human beings for clinical applications and intelligent robotic system designed for interactive rehabilitation.

## 2. Volumetric Neural Imaging

In the past few decades, one of the most exciting achievements in neural coding studies is volumetric functional imaging, which has enabled monitoring brain-wide neural activities at precise locations. In this section, we reviewed the recent advancements of the macroscale fMRI and the microscale/mesoscale optical microscopy, the two major classes of volumetric imaging techniques that have been used or have potential applications for rehabilitation. We also highlighted the application of volumetric imaging on neural network reconstruction, which is expected to have fundamental impact on rehabilitation.

### 2.1. Macroscale Imaging-Functional MRI

MRI opens a new window for observing the brain noninvasively. By measuring wave energy emitted from hydrogen atoms excited by a magnetic field, MRI can produce 3D images of anatomical structures or physiological status of a brain. In particular, fMRI, which measures the BOLD (blood-oxygen-level dependent) effect related to neural activities, provides a unique opportunity of recording activities of the whole human brain at a relatively high resolution. We can study neural encoding and decoding with fMRI, which presents individual voxels as the basic coding unit.

Encoding of fMRI predicts voxel-wise activities given certain stimuli. Kay et al. developed a visual encoding model with four components, including the set of stimuli, the features of stimuli, ROI in the brain, and the algorithm of model estimation [13]. This strategy can be adapted for encoding motion, where the first two components become the set of movements and the features of movements. Decoding of fMRI signal has been extensively studied. It maps voxel dynamics to external stimuli [14], motor behaviors [15], or even high-level cognitive states [16]. While general linear models have been successfully used to build mapping models, there are significant efforts underway for applying modern machine learning techniques [17], such as kernel methods [18], random forests [19], and manifold learning [20] for the same purpose.

Another exciting development in fMRI decoding is real-time fMRI (rtfMRI) [21], which takes advantage of online processing of fMRI images. Set up with decoding models trained offline, rtfMRI returns decoding results as interpretable feedback to the human subject within a short delay after acquisition. The process is fast enough for the subject to modulate the brain activity in the sense of real time. Powerful parallel processing frameworks, which are becoming more and more affordable nowadays, can further improve the decoding speed and potentially clear any bottlenecks in computational modules [22]. This creates an opportunity for building high degree-of-freedom noninvasive brain-machine interfaces using fMRI.

### 2.2. Microscale/Mesoscale Imaging-Optical Microscopy

At the microscopic/mesoscale level, the most common functional imaging technique is calcium imaging, which mainly uses small fluorescence dyes (e.g., fura-2), or genetically encoded fluorescence proteins (e.g., GCaMP), to measure the concentration fluctuation of free calcium ions in response to electrical signals. Calcium imaging can be used to measure a large population of neurons in vivo. Assisted by genetic engineering, it can measure specific types of neurons or neurons at specific locations. Recent breakthroughs demonstrated the real power of calcium imaging for studying the whole brain at the single cell level: Schrödel et al. recorded ~70% of head neurons of* C. elegans* using wide-field temporal focusing [23] and Ahrens et al. recorded more than 80% of all neurons of the larval zebrafish brain using light-sheet microscopy [4].

Studying neural coding with microscope imaging, however, is a relatively new research area, which has few original methods designed for special properties of calcium imaging data. Therefore, this presents a new opportunity and challenge to computer scientists and engineers to develop innovative computational algorithms and tools. One major challenge would be the big data problem, because scanning the whole brain at the microscopic scale will produce terabytes or even petabytes of data. Decomposing the data into tractable components and mapping them onto a low-dimensional feature space are a key to revealing unknown brain dynamics.

Although its direct clinical application is yet to be clear, microscopic imaging can revolutionize rehabilitation by providing the mechanism or theoretical support for designing optimal rehabilitation therapy. This will rely on animal studies, which are more accessible resources for understanding the human brain than the human brain itself. The relatively small scale of animal brains offers practical opportunities for understanding a complete nervous system at the single cell level. Due to the fundamental similarities among the motor systems of all animal species, we can build disease models on animals to study problems associated with rehabilitation. For example, Li et al. has developed a* C. elegans* model of ALS to evaluate the role of autophagy in the disease [24]. For the vertebrate species, there are mice models for studying motor axon regeneration and muscle reinnervation [25] and zebrafish models for studying brain disorders [26]. Another obvious advantage of setting experiments with animal models is the possibility of using a rich set of genetic tools for targeting specific neurons and manipulating neuron functions [27]. The recent development of optogenetics especially has allowed us to control neural activities more precisely than ever before. Combining optogenetics with calcium imaging will definitely provide a powerful tool for observing neural activities when activating or inhibiting a specific set of neurons [28].

### 2.3. Neural Network Reconstruction

The human brain is a highly dynamic network generating coordinated activities of billions of connected neurons. In this sense, neural rehabilitation is basically the recovery of impaired neural networks. Therefore, the advancement of rehabilitation techniques relies on how well we understand neural coding in the neural network, which in turn requires reconstructing functional network or connectome from real data.

Electrophysiological recordings have been used to reconstruct functional neural networks at different scales [29–31], but they have fundamental limitations in resolution or coverage (Table 1). For example, EEG/ECoG can only provide networks with a low spatial resolution due to their recording sites outside of the brain. On the other hand, while extracellular electrophysiological recordings of neuron activities have the single cell resolution, they are limited to a small subset of neurons with obscure identities.

To reconstruct a functional network with better resolution-scale trade-off, we must take advantage of volumetric functional imaging. A common practice for mapping the functional connectivity of human brains is to compute region correlations of the spontaneous fluctuation of the BOLD effect in resting-state fMRI imaging [32]. Various statistical correlation analysis approaches, such as clustering [33], independent component analysis [34], and Bayesian network [35], have been successfully applied. These approaches should also be applicable to microscopic functional imaging, which is currently at the stage of delivering whole brain data at single cell resolution.

**Figure 1:**
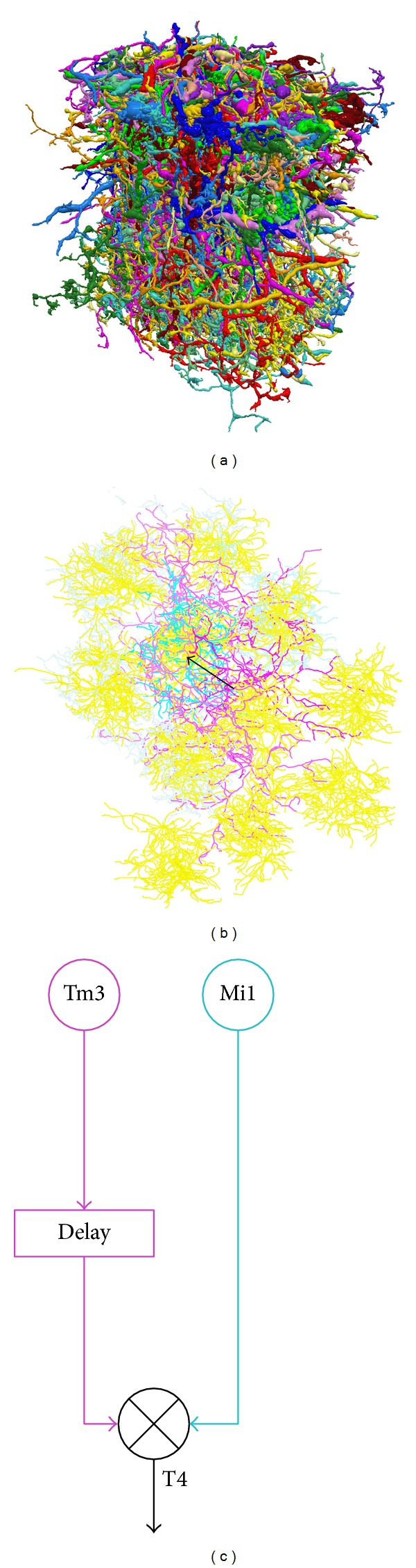
The motion detection circuit suggested by a connectome reconstructed from the* Drosophila* brain [39]. (a) Visualization of 379 neurons in the connectome, which is a part of the optical lobe; (b) the offset (black arrow) of the receptive fields computed from the circuit, which involves L1 (yellow), Tm3 (magenta), Mi1 (cyan), and T4 neurons, suggests a potential implementation of (c) the Hassenstein-Reichardt model.

We can also reconstruct neural networks anatomically and then infer the functional connectivity. This can be done by diffusion tensor imaging at the macroscopic scale [36], serial two-photon tomography at the mesoscale [37], and fluorescence microscopy [38] or electronic microscopy [39] at the microscopic scale. Although structural neural networks do not provide functional connections directly, they have rich clues about how information processing is implemented in the brain. For example, structural analysis of a circuit in the* Drosophila* optical lobe has, for the first time, shown that the brain computes the offsets of receptive fields to detect motion (Figure 1) [39]. How to integrate structural and functional neural networks would be a very interesting research topic, which holds promise for revealing fundamental rules of neural computation.

Undoubtedly, a better understanding of the structural and functional properties of the brain network will lead to more accurate simulations of the brain activities and behavior outputs. Recently, Eliasmith et al. reported a large-scale human brain model called “Spaun,” which can drive a physically modeled arm to draw pictures by following visual stimuli [40]. It is possible to use a similar framework to simulate motor behaviors given specific neuron degeneration or impaired conditions. These models can be further combined with realistic muscle-based locomotion models, such as those used in computer graphics [41], to assist in diagnosis or treatment planning.

## 3. Neural Coding in Brian Machine Interface (BMI)

Brain-machine interfaces exploit the spatial and temporal structure of neural activity of the brain to directly control a prosthetic device. This emerging field has been mainly inspired by the requirements of restoring interactions between the environment and the individuals with severe sensorimotor deficits through BMI-controlled systems. For example, a tetraplegic patient can feed herself with chocolate using a BMI-controlled robot arm [42].

### 3.1. BMI with Animal Models

Since the first experimental demonstration using the primary motor cortical signals of a rat to control a lever press [43], nonhuman primates have been utilized as ideal subjects for BMI studies [42–54] due to the similar functional brain structure as human beings [55], which enables the implantation of multiple electrode arrays in different motor cortical regions, and the better capability to perform complicated tasks than other animal models. Starting with a standard center-out movement task in primates, in which monkey's neural activities were found to be tuning to the directional movement [56], the ensemble of the neural firings could be used to predict more complicated arm movement in a 2D computer cursor control or 3D reaching and grasping, as well as the gripping force [47, 51, 57–59]. In 2008, Schwartz's group realized a real-time cortical control of a prosthetic robot arm for self-feeding without the real movement of the monkey's arm [53], which is an important step close to the later clinic BMI applications on human [42, 45].

**Figure 2:**
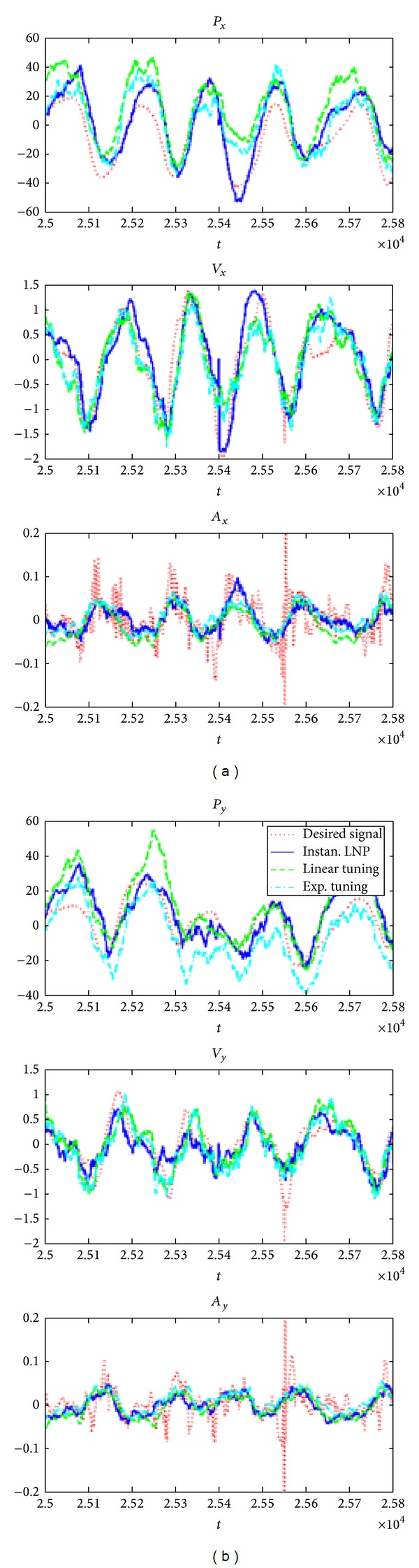
The reconstructed kinematics for a 2D reaching task by different tuning models from 185 neurons for 1000 testing samples (10 ms for each time instance) [67]. The left and right panels depict the reconstructed kinematics for the *x*-axis and the *y*-axis, respectively. The three rows of plots from top to bottom display the reconstructed position, the velocity, and the acceleration, respectively. In each subplot, the dotted red line indicates the desired signal, the solid blue line indicates the estimation using the proposed instantaneous LNP model, the dashed green line indicates the estimation using linear tuning, and the dot-dashed cyan line indicates the estimation using exponential tuning.

In a typical motor BMI framework, neuronal activity (local field potentials, single-unit activity, and multiunit activities) is synchronously collected from microelectrode arrays implanted into multiple cortical areas (primary motor cortex (M1), premotor cortex (PMA), supplementary motor cortex (SMA), primary somatosensory motor cortex (S1), posterior parietal area (PP), etc.) while the subjects are performing movement tasks. Several signal-decoding approaches have been applied to extract the functional relationship between the neural recordings and the subjects' kinematics. The decoder implements a model to predict movements and control a prosthetic robot arm or computer. The first issue in BMI decoding is the choice of the motor parameters, such as position, velocity, acceleration, gripping force, and even EMG signals, which are probably more promising for the patient to accept as the brain-muscular interfaces other than the stiff robot [46]. The second issue is to find a decoding algorithm to translate the cortical activities accurately. Many decoding methodologies use binned spike trains to predict movement based on linear or nonlinear optimal filters [50, 51, 54, 58, 60], but lack of further interpretation of the neurological dynamic tuning properties. Another method that derives the movement states probabilistically from the neural tuning model is to use a Bayesian formulation [61, 62]. It shares the parallel that the brain makes decisions based on prior knowledge [63]. As the binning on the spike does not exploit spike timing structure and may exclude rich neural dynamics in the modeling, the adaptive point process filtering methods have been developed to directly derive the kinematics from the spike trains [64, 65] with the modeling of the neural tuning properties to the instantaneous time instance and the connectivity among the neural ensemble [66, 67]. For example, Wang's work developed a novel, online, and encoding model that uses the instantaneous kinematic variables (position, velocity, and acceleration in 2D or 3D space) to estimate the mean value of an inhomogeneous Poisson model [67].  Figure 2 shows an implementation of an instantaneous tuning model in sequential Monte Carlo point process estimation based on spike timing, which provided statistically better kinematic reconstructions than the linear and exponential spike-tuning models in monkey.

**Figure 3:**
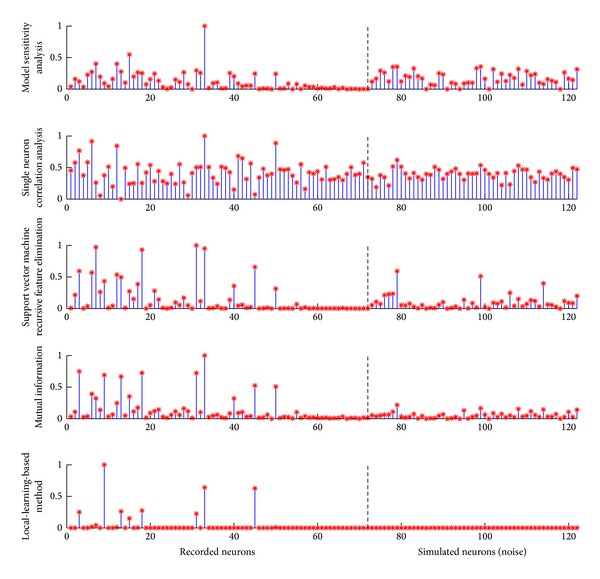
Distributions of the neuronal weights calculated by different methods, including single neuron correlation analysis, model sensitivity analysis, support vector machine recursive feature elimination, mutual information, and local-learning-based method. Neurons on the left side of the vertical dash line show real recordings. Neurons on the right are simulated and generated independently from the task. The five approaches show the different abilities of eliminating noisy neurons. The weights of the simulated neurons, learning from our proposed method, are all close to 0, while many of them are assigned with relatively large values by other methods [70].

**Figure 4:**
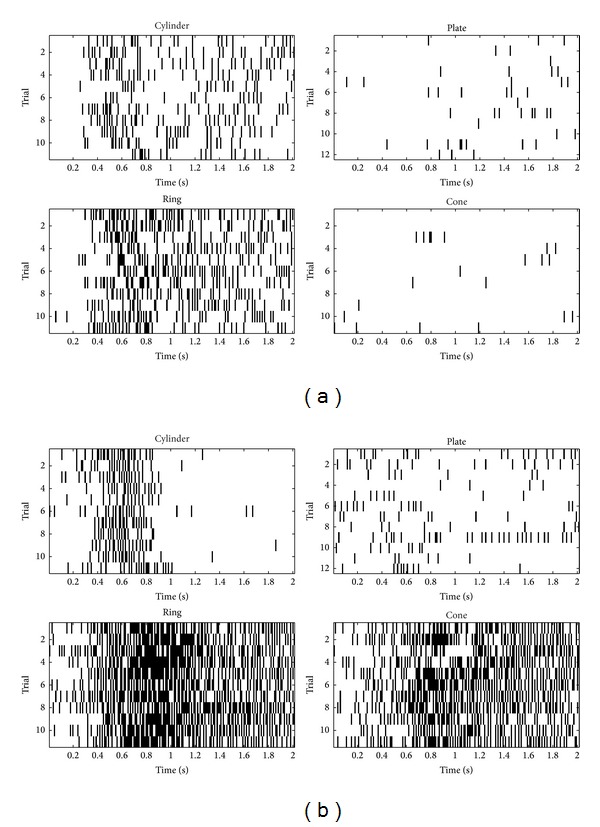
Temporal activities of the top two neurons corresponding to different grasping targets. The light was on at time 0; the object was grasped around time 1 s and was held until time 2 s. (a) The first neuron. (b) The second neuron. In each block, the grasping target in the upper left plot is a cylinder. The target in the upper right plot is a plate. The target in the bottom left plot is a ring. The target in the bottom right plot is a cone. Neuron 1 fires more frequently when the target objects were the cylinder and the ring, while much less for the plate and the cone. The activity of neuron 2 clearly distinguished the group of cylinder and plate and the group of ring and cone. Furthermore, neuron 2 ceased to fire around time 1 s, which separated the reaching period and the grasping period [70].

Aiming at the computational efficiency for the portable BMI devices, researchers also developed techniques to ascertain the neurons that relate the most to the movement task and gain better understanding of the individual neuron firing behavior [58, 68–70]. In Xu's work, a local-learning-based method was proposed to perform neuron selection for the gesture prediction in a monkey's reaching and grasping task [70]. The algorithm effectively ascertained the neuronal importance without assuming any coding model and provides a high performance with different decoding models. The method showed better robustness of identifying the important neurons with noisy signals presented, as shown in Figure 3. The ascertainment of the important neurons helped to inspect neural patterns visually associated with the movement task (Figure 4).

One important issue for clinical BMI application is to incorporate prosthesis devices into body representation and make it feel like the subject's own limb [71]. Introducing the sensory feedback including visual, auditory, and tactile cues, BMI therefore becomes a close loop system [72]. Although visual or auditory information is fed back to the subject in previous BMI designs [50, 51, 58], researchers investigated the possibility to embed peripheral tactile and proprioceptive signals into the prosthesis operation. O'Doherty et al. [48] implemented intracortical microstimulation techniques directly on the cortical area (S1) of the monkey in a BMI task, in which the monkey could distinguish 3 different targets due to the simulated sense of “touch” in the brain. However, arguments still remain whether the true sense of “touch” is reproduced or the monkey just learns the link between the targets and the electrical “tingling.” Other than intracortical microstimulation, optogenetic techniques become promising to active certain types of cells as stimulation to generate the peripheral tactile and proprioceptive feedback due to better temporal and spatial precision, easier manipulability, and less side effects [73]. Although study shows that mice could be guided to run in a circle by such techniques [74], there are few reports on monkey to appear difference in the behavior level using optogenetics as stimulation [75].

In the closed-loop BMI application, the subject needs to learn how to operate a BMI system using biofeedback. The neuroplasticity, induced by biofeedback, could help the subject adjust brain activity to better adapt to the system control over time [51, 76]. On the other hand, the adaptive decoders need to follow the nonstationary neural activities in order to improve the performance of BMI systems [77, 78]. The coadaptive BMI has later been presented as a novel architecture that goes beyond translational neural interface by merging with above two factors [51, 76, 79, 80]. Allowing brain and the intelligent decoder to adapt to each other during learning according to the task accomplishment, coadaptive BMI becomes attractive for the brain-controlled prosthesis in future clinical applications without requiring the real movements of the patients, for example, with tetraplegia.

### 3.2. Neural Coding for Human BMI

Similar to the coding methods used in animal models, the brain electrical signals used in BMI systems for human beings are mainly ECoG and scalp EEG. Scalp EEG (referred as EEG later) can be noninvasively detected from the skin surface according to the 10–20 system for positioning the electrodes, with the commonly adopted numbers of 32 and 64 channels for the whole brain recording, or according to 10–5 system for high-density EEG with 128 channels [81]. In comparison with another biosignal captured from the skin surface, EMG (50 *μ*V–10 mV), the amplitude of EEG (10–20 *μ*V) is much smaller and easily contaminated by head and neck muscle contractions, as well as artifacts, for example, eye blinks. EEG signals have been proposed for identifying neural instructions. For example, it has been widely known that the motion planning is associated with a decrease in EEG energy in the mu rhythm (8–12 Hz) over sensorimotor cortex, that is, event-related desynchronization (ERD), and after the execution of a motion there will be a rebound in the EEG power around 20 Hz, that is, event-related synchronization (ERS) [82]. EEG-based BMI systems have been successfully applied on external device control by people with severe motor disorders, such as spinal cord injury (SCI) and muscular dystrophies [83, 84]. However, most of them showed little effect on motor recovery for stroke rehabilitation as pointed out in the reviews of Belda-Lois et al. and Mattia et al. [85, 86]. One of the major reasons is that, different from subjects with an intact brain (e.g., SCI), individual stroke survivors have varied brain lesion sites and sizes, which increases the difficulty of recognizing the correct motion patterns for each. The second reason could be that stroke patients are suffered from involuntary muscle hypertonia more often than SCI patients [87], and it would introduce extra noises to the EEG signals recorded. The third reason might be associated with the method of whole brain EEG recording. Once the learning capability of a BMI algorithm is powerful and easy to converge to a classified pattern with redundant EEG channel information (or even with repeatable patterns of noise, like EMG), the effort from the other side of neuroplasticity in the brain will be weakened. The reported pattern recognition rates of BMI with EEG for stroke varied greatly (e.g., from 60% to 90% [88]), and usually are lower than those for SCI. Therefore, more effective and accurate neural indicators from the brain are needed for human BMI design, especially in stroke rehabilitation.

Different from the skin surface electrodes of EEG, electrode grids that acquire ECoG can be placed subdurally (i.e., below the dura mater) or epidurally (i.e., on top of the dura) directly on the surface of the brain (i.e., subdural recordings) or on top of the dura (i.e., epidural recordings). Hence, ECoG signals are mesoscale activity of ensembles of neurons, which lie in the continuum between microscale single-unit action potential firings recorded intracortically and to macroscale EEG from the surface of the scalp. In fact, the rapidly growing interest in ECoG is mostly due to its improved signal characteristics relative to the artifact prone EEG. Compared with EEG, ECoG has finer spatial resolution (mesoscale (millimeters) versus macroscale (centimeters)) [89–91], broader spectral range (0–500 Hz versus 0–40 Hz) [92], higher amplitude (i.e., 50–100 *μ*V versus 10–20 *μ*V) [93], and less vulnerability to movement artifacts [5, 93, 94]. Moreover, ECoG electrode grids, which are typically platinum electrodes 4 mm (2.3 mm exposed) in diameter and are configured in either a grid (e.g., 8 × 8 electrodes) or strip (e.g., 4 or 6 electrodes) configuration with an interelectrode distance of usually 10 mm, are far more likely to yield long-term functional stability [95–99] than intracortical electrodes, which induce complex histological responses that may impair neuronal recordings [100–102]. In fact, recent studies in primates demonstrated that the signal-to-noise ratio of ECoG signals is stable over several months [103]. Moreover these studies showed that cortical representations of three dimensional arm and joint movements that can be identified [7] and cortical control of three dimensional cursors were achieved [104] and maintained over several months.

However, since the placement of ECoG grid electrodes requires an invasive procedure (craniotomy and in most cases an incision to the dura), most ECoG-based human studies have recruited patients that were implanted as part of brain surgery to excise epileptic focus or mass lesion. Hence, most of the earlier ECoG-based studies often studied behaviors that were relevant to clinical evaluation of these patient populations, such as functional mapping of motor function. These early efforts culminated in the first comprehensive characterization of ECoG responses to visuomotor tasks in the late 1990s [105]. This has led to the appreciation of task-related modulations in high gamma (70–200 Hz) activity. The spatiotemporal patterns of these modulations are consistent sensorimotor function and its functional anatomy [106]. In recent years, however, ECoG has proved to be a vibrant recording technique for studying higher order functions [6, 107] and for brain-computer interfaces [108, 109] bringing together clinicians, neuroscientists, and engineers in the process. Moreover, epidural studies in animals [104, 110] corroborate the viability of epidural signals as a practical and less invasive signal modality, which can significantly reduce the risks of inflammations and complications.

The first use of ECoG as a practical and robust platform for translational applications beyond epileptology in human beings was demonstrated by Wang et al. [111] with an individual with tetraplegia caused by C4 level spinal cord injury. ECoG signals were recorded over the left sensorimotor cortex using a 32-contact high-density grid. The participant achieved robust volitional control of 3D cursor movement and a robotic arm. The participant was able to modulate his sensorimotor cortex with distinctive cortical activity patterns for different segments of the upper limb. The grid remained implanted for 28 days and did not cause any adverse effects. Another study by Hirata et al. showed control of a robotic arm in patients with moderate motor dysfunction due to stroke [112]. However, the effects of this training on rehabilitation in ipsilesional brain areas have not been recorded.

ECoG as a signal modality has also lent itself to uncovering potential signal features that could be exploited for hemispheric stroke. In recent years, there has been increased interest in how ipsilateral motor and motor-related areas activate in same-sided movements in both healthy and stroke-affected subjects [113–115]. These findings have motivated further explorations of whether ipsilateral activity in unaffected hemispheres could be used in neuroprosthetic applications for stroke-induced hemiparesis. Wisneski et al. [116] utilized ECoG recordings to comprehensively define ipsilateral physiology in motor-intact patients undergoing invasive monitoring. Electrocorticographic signals were recorded while the subjects engaged in ipsilateral and contralateral hand motor tasks. Ipsilateral hand movements were associated with low-frequency modulations (around 37.5 Hz) in premotor cortex ~160 ms before than activity related to contralateral hand movements. The authors therefore hypothesized that ipsilateral cortical activity is involved in motor planning (rather than execution). More recent studies [8, 117] have demonstrated that the ipsilateral cortical signals could be used to decode the direction of the joystick movement. Overall, these studies suggest that in motor-intact human subjects, ipsilateral activity during hand and arm movement is distinguishable from contralateral activity, is involved in planning rather than execution, and can be used as a viable control signal in BMIs [116]. Moreover, the fact that the premotor cortex control signal is in the low-frequency ranges highly suggests that ipsilateral (and contralesional) EEG signals could be used with patients with stroke for BMI control and possibly rehabilitation. In fact, functional imaging has demonstrated increased activity in the premotor cortices of motor-impaired stroke in unaffected hemispheres [118, 119]. This heightened activity could be a result of upregulation of motor planning due to the inability of executing the planned movement [116]. In a recent study Bundy et al. tested whether this heightened activity could be detected with an EEG-based BMI and converted into the desired action [88]. They recorded EEG signals from four chronic hemispheric stroke patients as they attempted real and imagined hand tasks using either their affected or unaffected hand. Low-frequency ipsilateral motor signals in the unaffected hemisphere, distinguishable from contralateral signals, were identified and subsequently used for a simple online BMI control task. They demonstrated that EEG signals from the unaffected hemisphere, associated with imagined movements of the affected hand, enabled stroke patients to control a cursor in one dimension. There is significant potential for this approach to be used as a novel tool for rehabilitation by slowly disengaging the unaffected hemisphere and engaging the affected hemisphere during BMI control.

## 4. Intelligent Rehabilitation Robots Based on EMG Coding

Intelligent rehabilitation robots usually refer to the systems that can interact with the voluntary motor intentions of a user. Besides the BMI technology introduced above, EMG-controlled robotic system is another choice for rehabilitation, mainly due to the easy-access of the signal from the skin surface of a muscle. In this part, an overview of the rehabilitation strategies in recent robots was introduced first, and it was followed with a review on some latest representative EMG-controlled rehabilitation robots and their clinical applications.

### 4.1. Rehabilitation Strategies in Robots

The aims of the treatment in neural rehabilitation are mainly to rebuild the lost sensorimotor functions due to nervous system injuries, such as stroke, and to minimize the related paretic symptoms. The recovery in the rehabilitation is a motor relearning process; that is, the lost functions can be regained and maximized by intensive and repeated voluntary practices [120, 121], and this concept has been applied in the traditional rehabilitation for decades.

Treatments in the rehabilitation are arduous processes. Training programs are usually time-consuming and labor-intensive for both the therapist and the patient in one-to-one manual interaction. In these situations, rehabilitation robots have acted as the assistance to therapists, providing safe and intensive physical training with repeated motions [122–128]. The most commonly reported motion types provided by developed rehabilitation robots are (1) continuous passive motion (CPM), (2) active-assisted movements, and (3) challenge-based movement. In treatments with continuous passive motion, the movements of the patient's limb(s) in the paretic side are guided by the robot system as the patient stays in a relaxed condition. This type of intervention was found to be effective in temporarily reducing muscular hypertonia and maintaining the flexibility of joints for stroke and spinal cord injury [129]; however, it contributed little to a permanent motor recovery in the central nervous system after stroke [129, 130]. In active-assisted robotic treatment (or interactive robotic treatment), the rehabilitation robot provides external assisting forces when the patient can not complete a desired movement independently [123, 131, 132]. In this type of physical training, the robot first needs to identify the motor intention from the patient and then provides the interactive assistance to the paralyzed limb. This type of training has been found to be more effective in motor improvement than CPM in stroke rehabilitation [129]. Robotic treatment with challenge-based movement can assign training tasks with varied difficulty levels [124, 133, 134], which is effective in promotion of voluntary efforts from the patient according to the recovery progress. Active-assisted and challenge-based robotic training can be combined in one treatment to achieve a maximized motor recovery, and the key to a successful rehabilitation is the accurate interpretation of the voluntary motor intention of a user.

### 4.2. EMG-Controlled Rehabilitation Robots

**Figure 5:**
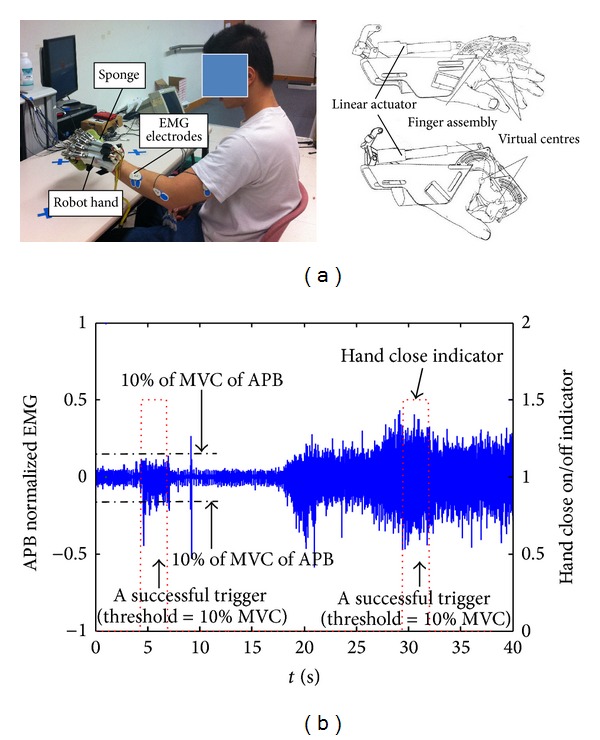
(a) The EMG-triggered robot hand for upper limb training and (b) the representative EMG-triggered cycles for hand close of the robot [10].

EMG is the electricity generated in muscles under the control of the nervous systems. When an action potential transmitted from a motor neuron axon to the muscle fibers, a motor unit action potential is evoked. In comparison with the amplitude of neuronal signals, EMG's amplitude is much higher (usually in millivolt) even when detecting from the skin surface [9]. Therefore, EMG is a favorable biosignal to represent a user's voluntary motor intention in robotic design. For interpretation of EMG in the real-time control of robots, there are basically two methods, triggered mode and continuous mode. In the triggered mode, EMG was used to initiate the movement of the robot, and after that, the robot would work in a CPM mode [10, 123, 132]. A preset threshold for detection of the onset of EMG can be used to trigger the motion of the robot, for example, the EMG-triggered hand robot for upper limb training after stroke introduced in the study of Hu et al., as shown in Figure 5(a) [10]. The robot hand could help a stroke patient perform the hand close/open motions triggered by the residual EMG detected from the abductor pollicis brevis (APB) in the paretic side for controlling the hand close and the extensor digitorum (ED) for the hand open.  Figure 5(b) shows the representative triggering cycles in the robot hand for hand close with the EMG from the APB muscle, where the triggering threshold was set at 10% of the EMG amplitude when conducting the maximal voluntary contraction (MVC) [10]. Once the real-time EMG amplitude was above the threshold and kept for 3 seconds, the robot hand would perform hand close motion with a constant angular velocity of 22°/s with the virtual center of the metacarpophalangeal (MCP) joints and 26°/s at that of proximal interphalangeal (PIP) joints. By using this EMG-triggered robot hand, a pilot clinical trial of upper limb training on ten subjects with chronic stroke was conducted, and each of the subjects received 20 training sessions with an intensity of 3–5 sessions/week. After the training, it was found that the robot hand assisted rehabilitation could significantly improve the finger functions and the muscle coordination in the whole upper limb [10].

**Figure 6:**
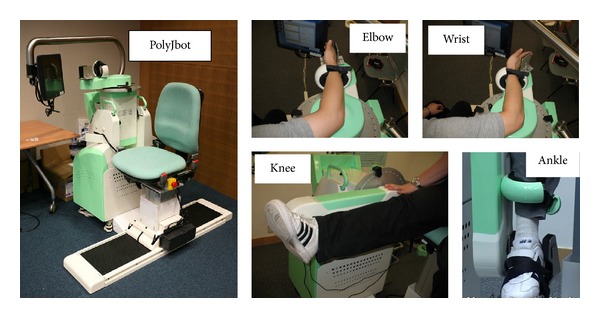
The continuous EMG-driven robot (PolyJbot) for joint training at the elbow, the wrist, the knee, and the ankle [130, 135, 136].

In a continuous EMG-driven robot system, the behaviors of the robot are controlled by the continuous variation of EMG, which requires the user to generate desired EMG patterns to instruct the movement of the robot [135], for example, the continuous EMG-driven robotic system (PolyJbot) developed for multijoint training by Tong's group shown in Figure 6 [130, 136, 137]. The robot can provide treatments on the elbow, the wrist, the knee, and the ankle with a continuous EMG-controlled algorithm. During the training, a subject needs to conduct joint extension and flexion by tracing a target cursor on the screen, and the robot will provide assistive torque to the joint, which is proportional to the EMG amplitude of a target muscle, for example, extensor carpi radialis (ECR) in wrist extension. In the algorithm design, the more muscle effort generated, the more assistive torque obtained with an attempt to maximize the voluntary effort during the training. In comparison with the training effects by robot-assisted CPM mode, the continuous EMG-driven mode could achieve more significant improvements in the release of muscle spasticity (i.e., hypertonia) and improvement of muscle coordination in the wrist joint, as well as in the shoulder/elbow part [130, 137]. Furthermore, the motor outcomes gained after the continuous EMG-driven robot-assisted training could be maintained for 3 months [130].

**Figure 7:**
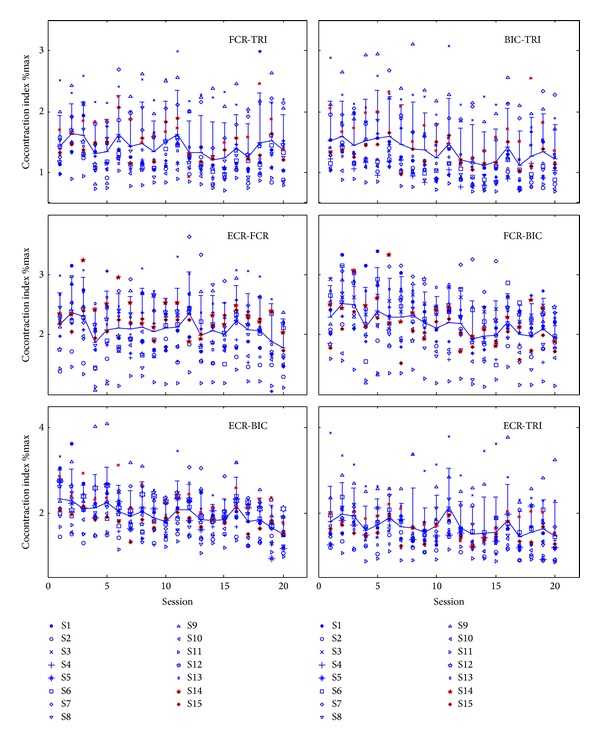
The cocontraction indexes between different muscle pairs during the continuous EMG-driven robot-assisted wrist training on 15 subjects with chronic stroke (S1–S15) [136]. FCR, flexor carpi radialis; ECR, extensor carpi radialis; BIC, biceps brachii; TRI, triceps brachii. The cocontraction index has a range from 0 to 1 and represents the extent of cocontraction phase of a pair of muscles, in comparison with the maximum value of 1. If both muscles contract at their maximum level at the same time, then the value will be 1; while if there is no cocontraction of the two muscles, the value will be zero [136].

EMG not only can be applied as the controlling signal in rehabilitation robots, but also has been applied on quantitative evaluation of the rehabilitation effects complementary to the subjective clinical assessment tools used in routine practice. For example, the coordination among muscles could be measured by EMG phasic change in muscle pairs [10, 130, 136–138]. The extent of the cocontraction phase was quantitatively evaluated by a cocontraction index (CI) between the EMG trials of two muscles. The CI values could be used to monitor the recovery progress in muscle coordination during the robot-assisted rehabilitation.  Figure 7 shows an example of the calculated CIs of the muscles in the upper limb during PolyJbot assisted poststroke wrist training in different sessions [137]. A decrease in the CI values usually was related to a release of muscle spasticity and more independent contraction of the muscle pair, that is, better coordination.

## 5. Future Prospects and Conclusions 

Modern neural rehabilitation heavily relies on the advances of neural computational techniques in diagnosis, treatment, and evaluation, which promise the future rehabilitation to be more automatic, economical, and convenient. Instead of receiving the treatments in hospitals or medical centers, future rehabilitation will be mainly home-based and subject-customized training with telecommunication for evaluation and follow-up to meet the fast growing market of home health care services [139]. To achieve this, further investigations are needed in neural coding techniques mainly in the following aspects.

1. Reduction of the calculation cost: high accuracy usually is sacrificed with the cost of calculation efficiency. Smart computational methods are needed to locate the areas of interest in neural imaging and highly relevant channels in BMI systems for individual subjects, with necessary accuracy for a real-time system. Currently, multichannel EEG systems are used for both invasive and noninvasive BMI systems, which are associated with large amounts of data to be explored. Effective channel selection methods with prioritized channel information should be useful to lower down the calculation cost, as some pioneers reported in the literature [140, 141]. More efforts are needed for the investigation of the long-term rehabilitation programs associated with the variation of neural plasticity in individuals, like persons after stroke who have varied brain lesions.
2. Comparative study between scalp EEG and ECoG: ECoG has higher resolution and signal quality than scalp EEG. However, the invasiveness of ECoG as a recording modality limits its usage. Modeling and source localization/projection studies could be helpful to investigate the relationships between ECoG and scalp EEG, which may lead to noninvasive EEG with needed resolution close to ECoG. For example, attempts have been made to compare the EEG and ECoG in persons with epilepsy [142]. However, more intensive investigations on the projection between EEG and ECoG are needed in other pathological and clinical applications in the future.
3. Rehabilitation effectiveness of BMI systems: although BMI-training systems have been proposed for patients with neural disorders, for example, stroke, the rehabilitation effects are still questioned [143]. Stroke patients could use the system with a high recognition rates. However, this may not directly lead to an improvement in the paralyzed limb functions [85, 86]. It is worthwhile to investigate whether redundant EEG channel information in the BMI system made the recognition task too easy to benefit the motor recovery.
4. Easy and reliable bioparameters in system control and evaluation of recovery: it is necessary to utilize objective and quantitative evaluation methods for monitoring recovery progress in rehabilitation. However, the operations of most of current robots are not easy for patients to use them at home without supervision. Training systems should be much simplified, especially on the detection of key bioparameters for system control and evaluation in the home-based devices.

## Figures and Tables

**Table 1:** Comparison of functional brain imaging methods.

	Temporal resolution	Spatial resolution	Advantage	Limitation
fMRI [144]	≥0.5 s	≥1 mm	Noninvasive whole brain imaging	Indirect measurement
Optical microscopy [23]	~0.2 s	~0.2 *μ*m	Single cell resolution	Unsuitable for human subject
Scalp EEG [145]	~1 ms	≥2 cm (128 channels)	Noninvasive whole brain recording	Low spatial resolution; signals are easily contaminated by noises (e.g., EMG, motion artifacts, etc.)
ECoG [95–99, 146]	~5 ms	~10 mm	Long-term and continuous recording	Invasive recording by attaching the electrode array on the surface of the brain
